# Natural language processing of electronic health records for early detection of cognitive decline: a systematic review

**DOI:** 10.1038/s41746-025-01527-z

**Published:** 2025-03-01

**Authors:** Ravi Shankar, Anjali Bundele, Amartya Mukhopadhyay

**Affiliations:** 1 Medical Affairs – Research Innovation & Enterprise, Alexandra Hospital, National University Health System, Singapore, Singapore; 2 Yong Loo Lin School of Medicine, National University of Singapore, Singapore, Singapore; 3 Division of Respiratory & Critical Care Medicine, Department of Medicine, National University Health System, Singapore, Singapore

**Keywords:** Health care, Medical research, Neurology

## Abstract

This systematic review evaluated natural language processing (NLP) approaches for detecting cognitive impairment in electronic health record clinical notes. Following PRISMA guidelines, we analyzed 18 studies (*n* = 1,064,530) that employed rule-based algorithms (67%), traditional machine learning (28%), and deep learning (17%). NLP models demonstrated robust performance in identifying cognitive decline, with median sensitivity 0.88 (IQR 0.74–0.91) and specificity 0.96 (IQR 0.81–0.99). Deep learning architectures achieved superior results, with area under the receiver operating characteristic curves up to 0.997. Major implementation challenges included incomplete electronic health record data capture, inconsistent clinical documentation practices, and limited external validation. While NLP demonstrates promise, successful clinical translation requires establishing standardized approaches, improving access to annotated datasets, and developing equitable deployment frameworks.

## Introduction

Cognitive impairment and dementia are rising global health priorities as populations rapidly age. Worldwide, around 55 million people live with dementia, a figure projected to reach 139 million by 2050^1^. Alzheimer’s disease (AD) is the leading cause, while milder impairments affect 15%–20% of those over 65^2,3^. The economic and social costs are immense, estimated at over US$1 trillion annually^4,5^.

Early detection is foundational for timely diagnosis, treatment, support, and planning that can improve patient and caregiver outcomes^6,7^. Even without disease-modifying drugs, early intervention may slow decline and enhance quality of life through risk factor modification, cognitive training, and care coordination^8,9^.

However, recognizing the initial signs of impairment remains challenging. Subtle changes in memory, language, and function are easily missed in brief clinical encounters and may elude routine cognitive screens^10^. Consequently, 40%–60% of dementia cases go undetected, delaying diagnosis by years^11,12^. The widespread adoption of EHRs offers new opportunities to mine the trove of clinical data for early clues. Unstructured narratives in clinical notes capture key details and nuances absent from structured fields^13^. Symptoms, exam findings, and concerns suggesting cognitive issues may be buried in this free text^14^.

However, sifting through the vast EHR to extract such signals is prohibitively time-consuming. Natural language processing (NLP), a branch of artificial intelligence, provides computational methods to make sense of unstructured text^15^. NLP techniques can automatically identify, extract, and classify relevant information from clinical notes based on linguistic patterns and clinical knowledge^16,17^.

Applying NLP to EHRs has shown promise for diverse applications such as case finding, decision support, and research^18^. An expanding literature has explored NLP of clinical notes to detect cognitive impairment, yet no systematic review has comprehensively analyzed the methods, findings, gaps, and implications across the spectrum of studies.

This systematic review aims to synthesize the research on using NLP methods on EHR clinical notes for detecting cognitive impairment and dementia in older adults. The key questions are:

1. What NLP techniques, target conditions, diagnostic criteria, and EHR data sources have been studied?
2. How accurate are NLP approaches in identifying cognitive impairment compared to reference standards?

## Results

**Fig. 1:**
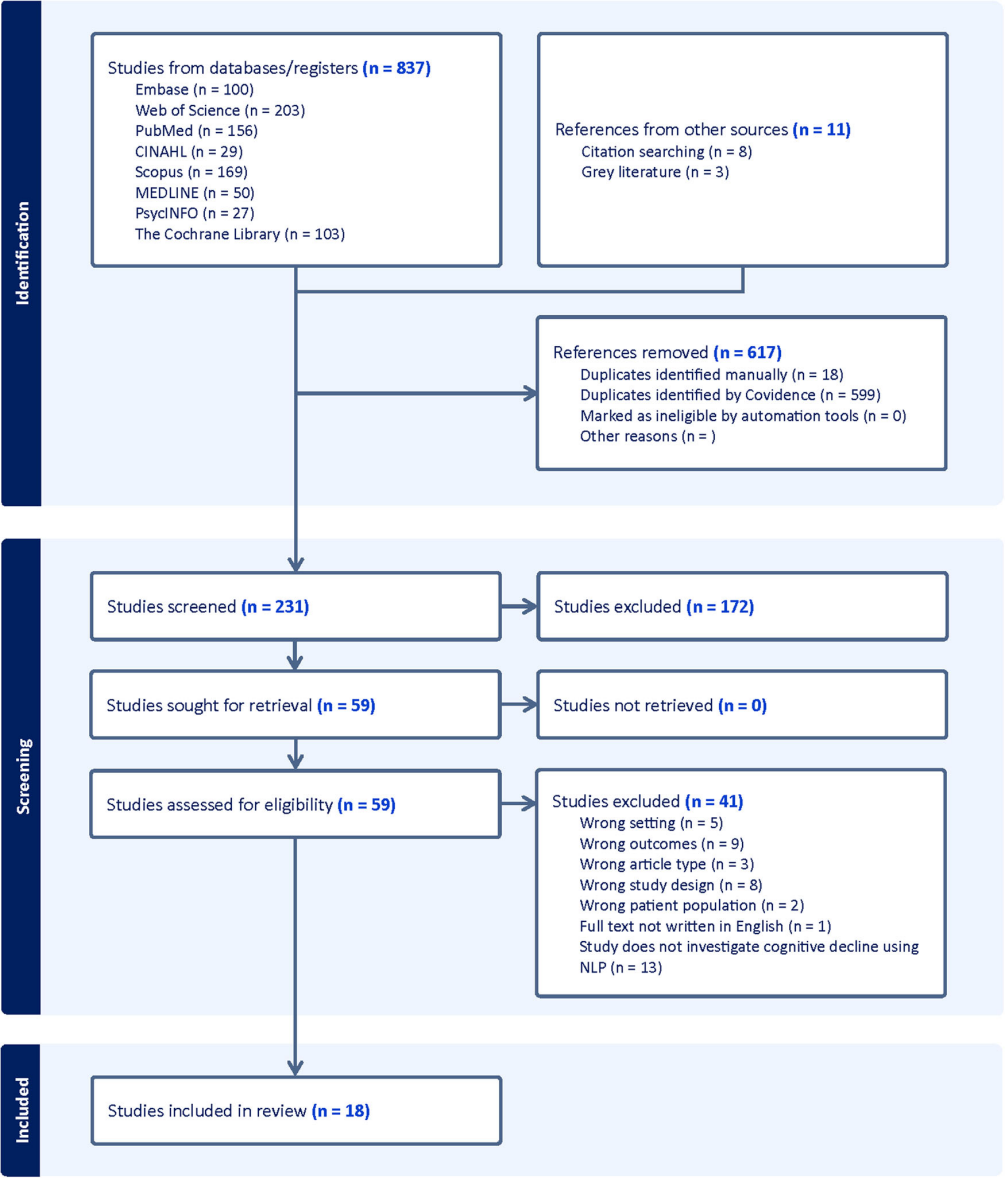
PRISMA flow diagram of study selection process. The systematic review identified 837 initial studies from database searches and 11 additional references from other sources. After removing 617 duplicates, 231 studies were screened. Full-text review of 59 articles resulted in 18 studies meeting all inclusion criteria for final analysis.

The final analysis included 18 studies that met all inclusion criteria and specifically investigated cognitive decline using natural language processing of electronic health record clinical notes in older adults (Fig. 1). We narratively synthesized study characteristics, NLP methods, key findings, challenges, and implications.

### Study characteristics

The 18 studies were published from 2020 to 2024 and conducted primarily in the USA (*n* = 14, 78%), UK (*n* = 3, 17%), and Canada (*n* = 1, 6%). Most used a retrospective cohort design (*n* = 14, 78%) in academic health systems (*n* = 9, 50%), integrated delivery networks (*n* = 3, 17%), and dementia/mental health services (*n* = 4, 22%). Sample sizes ranged from 199 to 535,814 (median 11,106). Participant characteristics are detailed in Table 1.

**Table 1:** Study characteristics, population demographics, and additional characteristics

Study	Country	Study design	Setting	Sample size	Mean age (years)	% Female	% Non-White	Cognitive status	Comorbidities
Zolnoori et al.^25^	USA	Retrospective cohort	Home healthcare	24,874	76.6	65.2%	33.7%	Not reported	Not reported
McCoy et al.^31^	USA	Retrospective cohort	Academic medical centers	535,814	53.3	59.1%	23.6%	No dementia	Stroke 3.5%
Prakash et al.^19^	USA	Retrospective cohort	Academic health system	9115	78	60%	Not reported	43.8% CI	Depression 68%, HTN 47.5%
Panahi et al.^26^	USA	Retrospective cohort	VA Health System	200	56	11%	28.5%	Not reported	Depression 68%, TBI 43.5%
Penfold et al.^21^	USA	Retrospective cohort	Primary care	4185	64.9	59.4%	10.2%	29.8%–50% MCI	Not reported
Noori et al.^29^	USA	Diagnostic study	Academic health system	2166	52.6–76.5	53%	69.1%	Not reported	Not reported
Oh et al.^20^	USA	Retrospective study	Academic specialty clinics	2680	79.1 (median)	61.3%	17.6%	100% AD	Not reported
Wang et al.^38^	USA	Retrospective cohort	Academic medical centers	162,069	Not reported	40.9%	Not reported	Normal cognition	Not reported
Du et al.^24^	USA	Retrospective study	Academic health system	6945	≥50	Not rep.	Not reported	Not reported	Not reported
Wang et al.^23^	USA	Diagnostic study	Academic health system	2166	76.3	53.1%	Not reported	Not reported	Not reported
Maclagan et al.^28^	Canada	Retrospective cohort	Primary care	44,674	74.6–80.3	55%–59%	Not reported	1.2% dementia	HTN 53.8%, depression 20.5%
Kent et al.^30^	USA	Retrospective cohort	Integrated health system	241,050	64.9	61.3%	57.2%	Not reported	HTN 60.4%, CAD 10.7%
Gibson et al.^27^	UK	Retrospective cohort	Mental health services	1425	72.3	61.3%	41.6%	47.1% CI	Not reported
Tyagi et al.^22^	USA	Retrospective cohort	Academic health system	16,428	73	46.8%	9.3%	Not reported	Not reported
Hussein et al.^33^	USA	Cross-sectional	Primary care and pulmonary	199	55–71+	74.9%	85.4%	40% MMSE < 24	Not reported
Bishara et al.^34^	UK	Retrospective cohort	Mental healthcare	14,093	79.8	60.7%	24.4%	MMSE 18.6	Not reported
Al-Harrasi et al.^36^	UK	Retrospective cohort	Dementia care services	11,106	80.6	62.7%	24.6%	100% dementia	Not reported
Purks et al.^35^	US/Canada	Cross-sectional	Online study	25,192	Median 67	45%	7%	32% self-reported CI	Depression 37%

*AD* Alzheimer’s disease, *CI* cognitive impairment, *CRIS* Clinical Record Interactive Search, *EHR* electronic health record, *HTN* hypertension, *MCI* mild cognitive impairment, *MMSE* Mini-Mental State Examination, *MD* medical doctor, *Rep.* reported, *TBI* traumatic brain injury, *UK* United Kingdom, *US/USA* United States/United States of America, *VA* Veterans Affairs.

### NLP methods

Twelve studies (67%) used rule-based NLP, five (28%) used machine learning, and three (17%) used deep learning (Table 2). Rule-based systems typically combined keyword searches, regular expressions, and clinical terminologies to extract relevant signs and symptoms. Machine learning models were trained on expert-annotated notes to classify cognitive status. Deep learning leveraged neural networks pretrained on large corpora and fine-tuned for the specific task.

**Table 2:** EHR data sources, NLP methods, and machine learning performance, and outcomes

Study	EHR system and time period	Note types	NLP approach and techniques	Knowledge sources	NLP performance	ML algorithm and validation	Performance metrics (AUC, sensitivity, specificity, F1)	Comparator (reference standard)	Predicted outcome	Outcome definition
Zolnoori et al.^25^	Allscripts, 2010-2012	Visit notes, care coord notes	Rules-based: Keyword matching, semantic	UMLS	Not reported	Various, Stratified 10-fold	AUC 0.76, Sens 0.75, Spec Not rep., F1 0.63	Claims data	Dementia	CCW flag
McCoy et al.^31^	Not reported, 2005–2017	Hospital discharge notes	Rules-based: cognitive symptom score	Custom term lists	Not reported	Cox, competing, split	AUC 0.71, Sens 0.65, Spec 0.66, F1 Not r.	ICD-9/10 codes	All-cause dementia	Structured diagnosis codes
Prakash et al.^19^	Epic, 2014–2019	All (> 65,000 notes)	Rules-based: pattern matching	Custom ontology	88% success rate	N/A (rule-based), N/A	AUC N/A, Sens 0.88–0.90, Spec 0.80–0.98, F1 0.83–0.92	Expert annotation	Mild/Mod-Severe AD	Severity terms
Panahi et al.^26^	VA system, 2001–2019	1195 selected note title types	Rules-based: concept extraction	Custom ontology	Not reported	Random forest, not reported	AUC 0.74, Sens 0.67, Spec 0.81, F1 Not r.	ICD-9 331.1	FTD	Positive predictive value 0.95
Penfold et al.^21^	Epic, 2004–2015	Primary care and behavioral health	Rules-based: MMSE score extraction	Not reported	Not reported	Logistic, XGBoost, Split	AUC 0.67, Sens 0.017, Spec 0.997, F1 Not r.	Validated HAD algorithm	MCI	Claims based
Noori et al.^29^	Epic, 2010–2020	All	Rules-based: keyword matching	Custom lists	Cohen’s *κ* = 0.67	N/A (manual rev.), N/A	AUC N/A, Sens N/A, Spec N/A, F1 N/A	Expert annotation	Normal, CI, undetermined	Manual chart review
Oh et al.^20^	Allscripts TouchWorks, 2013–2018	Not specified	Rules-based: pattern matching	UMLS, custom	Precision 0.30–1.0	N/A (rule-based), N/A	AUC N/A, Sens Not rep., Spec Not rep., F1 0.65–1.0	ICD codes	AD	F00 and others
Wang et al.^38^	Mass General Brigham DW, 2000–2021	Not specified	Deep learning: not specified	Not reported	Not reported	Cox, N/A	AUC N/A, Sens N/A, Spec N/A, F1 N/A	ICD codes/Problem lists	MCI/dementia	Structured diagnoses
Du et al.^24^	Not specified, Up to 2019	Not specified	Deep learning: large language models	Not reported	Not reported	Ensemble, Split	AUC N/A, Sens 0.94, Spec Not rep., F1 0.92	2019 MCI diagnosis	MCI	G31.84
Wang et al.^23^	Mass General Brigham DW, Up to 2019	Not specified	Deep learning: ClinicalBERT	MIMIC-III	Not reported	Deep learning, 5-fold CV	AUC 0.97, Sens Not rep., Spec Not rep., F1 Not r.	Expert annotation	CI, Severity	Manual chart review
Maclagan et al.^28^	EMRPC (unspecified), Up to 2016	Progress notes, consult notes	Rules-based: concept matching	Custom lists	Not reported	Logistic, 5-fold CV	AUC N/A, Sens 0.72, Spec 0.998, F1 0.77	Validated claims algorithm	Dementia	≥1 hosp and/or ≥3 MD visits or ChEI Rx
Kent et al.^30^	Kaiser Permanente, 2009–2019	Radiology reports (CT/MRI)	Rules-based: information extraction	Custom, MedTaggerIE	F1 0.90–0.91	Cox, competing, split	AUC N/A, Sens N/A, Spec N/A, F1 N/A	ICD-9/10 codes	Dementia	Codes + cognitive med or 2nd code
Gibson et al.^27^	CRIS (custom), 2008–2021	All	Rules-based: not specified	Custom lists	Not reported	Cox, N/A	AUC N/A, Sens N/A, Spec N/A, F1 N/A	ICD-10, McKeith criteria	Probable DLB	≥2 core features
Tyagi et al.^22^	Not specified, Not specified	Clinician notes	Rules-based: concept matching	SNOMED CT	Not reported	RF, deep learning, 90/10 split	AUC 0.98, Sens 0.91, Spec 0.96, F1 0.93	Not specified	AD, HC	Not specified
Hussein et al.^33^	Not specified, Not specified	Progress notes, discharge summaries	Rules-based: concept matching	SNOMED CT	Not reported	Logistic, RF, 100-fold CV	AUC N/A, Sens 0.95, Spec 1.00, F1 0.98	MMSE < 24	CI	Validated cut-off
Bishara et al.^34^	CRIS (custom), 2007–2015	Not specified	Rules-based: MMSE/medication extraction	Custom, GATE	Not reported	Not specified, Not specified	AUC N/A, Sens N/A, Spec N/A, F1 N/A	Not applicable	Cognitive trajectory, medication use	MMSE scores, exposure groups
Al-Harrasi et al.^36^	CRIS (custom), 2007–2015	Not specified	Rules-based: information extraction	Custom, ADEPt	Precision 0.89, Recall 0.88	Not specified, not specified	AUC N/A, Sens N/A, Spec N/A, F1 N/A	ICD-10	AD, Vasc., mixed	F00, F01
Purks et al.^35^	N/A (online), Up to 2020	Self-reported data	Rules-based, ML: not specified	Not reported	Accuracy ≥96%	N/A (rule-based), N/A	AUC N/A, Sens N/A, Spec N/A, F1 N/A	Self-report	FTD phenotypes	Categorized symptoms

*AD* Alzheimer’s disease, *ADEPt* Adverse Drug Event Annotation Pipeline, *AUC* area under the receiver operating characteristic curve, *CAD* coronary artery disease, *CCW* Chronic Condition Warehouse, *ChEI* cholinesterase inhibitor, *CI* cognitive impairment, *CRIS* Clinical Record Interactive Search, *CT* computed tomography, *CV* cross-validation, *DLB* dementia with Lewy Bodies, *DW* data warehouse, *EHR* electronic health record, *EMRPC* electronic medical records primary care, *FTD* frontotemporal dementia, *GATE* General Architecture for Text Engineering, *HAD* Health Administrative Data, *HC* healthy control, *HTN* hypertension, *ICD* International Classification of Diseases, *MCI* mild cognitive impairment, *MIMIC* Medical Information Mart for Intensive Care, *ML* machine learning, *MMSE* Mini-Mental State Exam, *MRI* magnetic resonance imaging, *NLP* natural language processing, *RF* random forest, *Rx* prescription, *SNOMED CT* Systematized Nomenclature of Medicine - Clinical Terms, *TBI* traumatic brain injury, *UMLS* Unified Medical Language System, *Vasc.* vascular.

Common NLP targets included named entity recognition, concept extraction, and document classification. The most frequent note types were progress notes, consult notes, and discharge summaries. NLP results were often combined with structured EHR data (e.g. diagnostic codes, medications, labs).

### NLP performance

Across the 18 reviewed studies, NLP models demonstrated robust performance in identifying various cognitive decline phenotypes, including mild cognitive impairment (MCI), Alzheimer’s disease (AD), vascular dementia, frontotemporal dementia (FTD), and dementia with Lewy bodies (DLB). The primary NLP performance outcomes are summarized in Table 2. For detecting cognitive impairment, sensitivities ranged from 0.65 to 0.95 (median 0.88, IQR 0.74–0.91), and specificities ranged from 0.66 to 1.00 (median 0.96, IQR 0.81–0.99). Area under the receiver operating characteristic curve (AUC) values, reported in 6 studies, varied from 0.67 to 0.98 (median 0.86).

### Performance Evaluation

Performance varied systematically across cognitive phenotypes and clinical contexts. NLP systems achieved the highest accuracy for established dementia diagnoses using standardized criteria (median sensitivity 0.91, specificity 0.97), while performance was more modest for detecting mild cognitive impairment and early-stage disease (median sensitivity 0.76, specificity 0.89). This pattern likely reflects the more subtle and variable manifestations of early cognitive decline in clinical documentation.

Several studies focused on detecting AD and related dementias using rule-based NLP systems. Prakash et al.^19^ developed a rule-based algorithm using regular expressions and expert-curated lexicons to extract AD severity information from unstructured clinical notes in an academic health system. The algorithm achieved accuracies over 91% for identifying mild vs. moderate/severe AD. Similarly, Oh et al.^20^ employed pattern matching with curated ontologies to extract AD phenotypes from specialty clinic notes, reporting F1 scores ranging from 0.65 to 1.00 across various clinical features. These rule-based approaches leveraged domain expertise to achieve high precision but may struggle with linguistic variability.

Traditional machine learning approaches showed promise for detecting MCI, though with more variable results. A study by Penfold et al.^21^ compared multiple classifiers (logistic regression, random forest, gradient boosting) trained on features extracted from primary care notes using pyTAKES. While achieving high specificity (99.7%), their best model had limited sensitivity (1.7%), highlighting common challenges in MCI detection including incomplete cognitive assessments and imperfect reference standards. Other machine learning studies achieved more balanced performance through careful feature engineering and ensemble methods. In another study, Tyagi et al.^22^ trained logistic regression, multilayer perceptron, and random forest models on SNOMED CT concepts extracted from unstructured clinician notes. The random forest model achieved a sensitivity of 0.95 and a specificity of 1.00 for classifying AD and healthy controls.

Deep learning architectures, particularly transformer-based models like BERT, have showcased state-of-the-art performance for AD detection. Wang et al.^23^ fine-tuned a ClinicalBERT model on EHR notes from an academic health system, achieving an AUC of 0.997 and an area under the precision-recall curve (AUPRC) of 0.929 on a held-out test set. The model could identify early signs of cognitive decline up to 4 years before the initial MCI diagnosis. Du et al.^24^ used an ensemble of BERT models and traditional machine learning to detect MCI from EHR notes, reporting a sensitivity of 94.2% and an F1 score of 92.2%. These transformer-based architectures can capture complex semantic relationships in clinical text, enabling more accurate detection of subtle cognitive changes.

### Comparative Analysis of NLP Methodologies

The comparative analysis of NLP methodologies revealed distinct trade-offs. Rule-based systems excelled in precision and interpretability but required substantial expert input and showed limited generalizability. Machine learning approaches offered greater adaptability but depended heavily on training data quality. Deep learning models achieved superior performance but with increased computational demands and reduced transparency.

For instance, Zolnoori et al.^25^ used a combination of keyword matching and semantic analysis based on the Unified Medical Language System (UMLS) to extract dementia-related symptoms and risk factors from home healthcare assessments, achieving an AUC of 0.76 for predicting dementia risk. While rule-based methods can achieve high precision, they often require extensive expert knowledge and may struggle with the variability and ambiguity of natural language.

Machine learning-based NLP models automatically learn to recognize patterns from annotated training data. Penfold et al.^21^ compared the performance of various classifiers for using bag-of-words and term frequency-inverse document frequency (TF-IDF) features. However, these models typically rely on sparse vector representations of text that may fail to capture the rich semantic and syntactic relationships between words.

Deep learning architectures have achieved state-of-the-art performance on various NLP tasks. The models developed by Wang et al.^23^ and Du et al.^24^ could learn contextual word embeddings that capture the nuanced meanings of terms in different contexts, enabling a more robust understanding of the clinical narrative. However, their performance gains come at the cost of increased computational complexity, reduced interpretability, and the need for large-scale annotated datasets.

For specific dementia subtypes, NLP models achieved promising results in identifying FTD and DLB. Panahi et al.^26^ developed a rule-based system using a custom ontology to extract symptoms from veterans’ health records. The NLP pipeline achieved an 88% success rate in identifying FTD cases, which were then clustered using a random forest classifier. Gibson et al.^27^ focused on DLB, extracting core diagnostic features using NLP algorithms applied to mental health records. Having two or more core features at psychosis onset predicted probable DLB with a specificity of 81.2%, sensitivity of 66.7%, and AUC of 0.74.

### Healthcare Setting Impact on NLP Performance

Healthcare setting significantly impacted NLP performance, reflecting differences in documentation practices and patient populations. Primary care studies generally showed lower sensitivity but maintained high specificity—exemplified by Maclagan et al.’s^28^ work using SNOMED CT-based machine learning (sensitivity 71.5%, specificity 99.8%). This pattern suggests utility for screening but highlights challenges in detecting subtle cognitive changes in primary care documentation. In contrast, specialty clinics and research cohorts often had more detailed data and formal diagnostic assessments. Noori et al.^29^ developed an NLP annotation tool for cognitive phenotyping in an academic medical center, demonstrating substantial agreement (Cohen’s *κ* = 0.67) with expert chart review.

Large integrated health systems and national databases enabled population-scale analyses. Kent et al.^30^ used NLP to extract cerebrovascular disease from neuroimaging reports in over 240,000 patients, finding strong associations with future dementia risk. McCoy et al.^31^ developed a cognitive symptom score from discharge notes to stratify dementia risk in a cohort of over 500,000. While these studies leveraged the breadth of EHR data, they often relied on diagnostic codes as imperfect reference standards.

Across all settings, integrating NLP results with structured EHR data (e.g., demographics, medications, diagnoses) consistently improved performance compared to either data source alone. For instance, Tyagi et al.^22^ combined SNOMED CT concepts with machine learning to classify AD and healthy controls, reporting an AUC of 0.98, sensitivity of 0.91, and specificity of 0.96. This multi-modal approach helped overcome limitations of individual data sources while leveraging complementary signals.

### Feature Analysis and Evaluation Approaches

Performance evaluation approaches also varied significantly across studies. While most reported standard metrics (sensitivity, specificity, AUC), few conducted comprehensive error analyses or assessed performance stability across subgroups. Studies using cross-validation generally showed more robust results than those using simple train-test splits. External validation was rare but revealed important generalizability challenges.

The granularity and specificity of the extracted symptomatic features varied considerably across studies. While some focused on a broad set of cognitive and functional domains^25^, others targeted specific neuropsychiatric symptoms and biomarkers associated with particular dementia subtypes^20,27^. Zolnoori et al.^25^ focused on a broad set of 39 cognitive and functional domains organized into 7 Research Domain Criteria (RDoC), while Oh et al.^20^ targeted specific neuropsychiatric symptoms and biomarkers associated with Alzheimer’s disease. Gibson et al.^27^ extracted features related to the core clinical criteria for dementia with Lewy bodies, including visual hallucinations, fluctuating cognition, rapid eye movement sleep behavior disorder, and parkinsonism. The choice of features was often guided by clinical expertise, literature review, and the availability of annotated data. Few studies systematically evaluated the predictive power of individual features or feature combinations for early diagnosis of cognitive decline. Prakash et al.^19^ reported that the most informative features for their rule-based algorithm were memory complaints, repetitive questioning, and temporal disorientation. Du et al.^24^ found that the combination of BERT-extracted features and structured EHR data outperformed either data source alone, highlighting the importance of multi-modal data integration.

Moreover, the temporal dynamics of symptomatic features were rarely explored, despite their potential to provide valuable insights into the trajectory of cognitive decline. Wang et al.^23^ used a time-dependent Cox proportional hazards model to assess the association between NLP-derived depressive symptoms and incident MCI or dementia. Penfold et al.^21^ incorporated time-dependent features, such as the persistence of neuropsychiatric symptoms and healthcare utilization patterns, into their machine learning models, achieving an AUC of 0.84 for predicting 2-year risk of dementia.

### Risk of bias assessment

Two reviewers independently appraised studies with the Quality Assessment of Diagnostic Accuracy Studies-2 (QUADAS-2) tool for patient selection, index test, reference standard, and patient flow^32^. Discrepancies were resolved by discussion.

The risk of bias and applicability concerns are represented by colored circles, with green indicating low risk/concern, yellow indicating unclear risk/concern, and red indicating high risk/concern. The comments column provides a brief summary of the key methodological strengths and limitations for each study (Table  3).

**Table 3:** Quality assessment of included studies using QUADAS-2

First author	Risk of bias	Applicability concerns	Comments
Zolnoori et al.^25^			Clear inclusion criteria, well-described index test, and reference standard.
McCoy et al.^31^			Large cohort, index test not fully described.
Prakash et al.^19^			Patient selection criteria not fully described.
Panahi et al.^26^			Limited to veterans population.
Penfold et al.^21^			Reference standard may not capture all cases.
Noori et al.^29^			Reference standard not fully described.
Oh et al.^20^			Unclear if all patients received reference standard.
Wang et al.^38^			Index test not fully described.
Du et al.^24^			Patient selection, index test, and reference standard not fully described.
Wang et al.^23^			Clear inclusion criteria, well-defined index test and reference standard.
Maclagan et al.^28^			Reference standard may not capture all cases.
Kent et al.^30^			Clear inclusion criteria, well-defined index test and reference standard.
Gibson et al.^27^			Limited to mental health service population, index test not fully described.
Tyagi et al.^22^			Reference standard may not capture all cases.
Hussein et al.^33^			Non-consecutive sampling, potential selection bias.
Bishara et al.^34^			Limited to mental health service population, index test and reference standard not fully described.
Al-Harrasi et al.^36^			Limited to dementia specialty services, unclear if all patients received reference standard.
Purks et al.^35^			Self-selected online cohort, potential selection bias, reference standard may not accurately capture cognitive status.

## Discussion

This systematic review provides a comprehensive synthesis of the rapidly evolving evidence on using NLP applied to EHR notes for detecting cognitive impairment in older adults. The 18 included studies, representing over 1 million patients across diverse healthcare settings, demonstrate the potential of NLP to identify diagnostically relevant information from unstructured clinical text. The reviewed studies employed various NLP techniques, including rule-based algorithms, traditional machine learning, and state-of-the-art deep learning, to extract and classify signs, symptoms, and risk factors of cognitive decline. The performance metrics reported in these studies are promising, with most models achieving high sensitivity (median 0.88, IQR 0.74–0.91) and specificity (median 0.96, IQR 0.81–0.99) in detecting cognitive impairment against reference standards. Deep learning approaches showcased particularly impressive results, with AUROCs reaching up to 0.997 in some cases. The strong predictive performance across various modeling strategies highlights the feasibility of using NLP to surface subtle indicators of cognitive decline that may be missed in routine care, thus enabling earlier detection and intervention.

Despite the promising results, several challenges and limitations were identified across the studies which should be considered when interpreting their findings. The heterogeneity in reported metrics and limited details on error analysis preclude drawing definitive comparisons between NLP techniques. The reliance on imperfect reference standards, such as diagnostic codes or brief cognitive screening tools, may have introduced bias or noise in the labels used for model training and evaluation. The most frequent limitations were the retrospective nature of studies and the use of a single healthcare system (*n* = 14, 78%), limited adjustment for potential confounders (*n* = 12, 67%), incomplete EHR data capture (*n* = 10, 56%), and reliance on diagnostic codes as the reference standard (*n* = 9, 50%). These limitations were particularly evident in studies like Tyagi et al.^22^ and Gibson et al.^27^, where the single-center nature of the research and limited diversity in study populations raised concerns about generalizability. Other notable limitations included potential documentation biases (*n* = 8, 44%), lack of access to neuroimaging data (*n* = 4, 22%), and potential algorithmic biases related to factors such as race and education (*n* = 4, 22%). Less common but still important limitations were small sample sizes (*n* = 3, 17%), limited generalizability due to specific study populations (*n* = 3, 17%), and lack of external validation (*n* = 3, 17%). Additionally, few studies assessed model performance across different sociodemographic groups, leaving open the possibility of algorithmic bias and disparity propagation. Future studies should aim to address these limitations by utilizing diverse data sources, carefully adjusting for confounding factors, and validating models in external populations to improve the robustness and generalizability of NLP-based approaches for early detection of cognitive decline.

The synthesis also revealed a notable scarcity of research on the real-world implementation and impact of NLP-based tools for cognitive impairment detection. Out of the 18 included studies, only 4 (22%) were conducted in real-world clinical settings: Zolnoori et al.^25^ in a home healthcare setting, Penfold et al.^21^ and Maclagan et al.^28^ in primary care settings, and Kent et al.^30^ in an integrated health system. Studies like Du et al.^24^ and Prakash et al.^19^ highlighted the need for standardized approaches, improved access to annotated datasets, and the development of equitable deployment frameworks. For example, Hussein et al.^33^ achieved impressive performance metrics (sensitivity 0.95, specificity 1.00) but noted significant barriers to clinical implementation including the need for robust validation and concerns about algorithmic bias. These studies provided valuable insights into the potential of NLP in actual healthcare environments, but further work is needed to assess the feasibility, acceptability, and effectiveness of these tools when integrated into clinical workflows. Bishara et al.^34^ and Purks et al.^35^ emphasized the importance of standardized approaches for clinical integration. Similarly, Maclagan et al.^28^ underscored the need for seamless electronic health record (EHR) integration, minimal disruption to clinical workflows, and robust validation processes. The studies conducted in academic or research settings also highlighted the need for more real-world implementation and evaluation. This gap underscores the need for future work to move beyond model development and validation to grapple with the complex sociotechnical challenges of deploying AI in healthcare contexts.

Other key issues discussed in these studies were provider acceptance, model interpretability, data privacy, and health system impacts. Future studies should focus on addressing issues such as data quality, interoperability, user adoption, and the impact on patient outcomes and care processes. By conducting more research in diverse clinical settings, the field can better understand the translational potential of NLP-based approaches and develop strategies to bridge the gap between research and practice in the early detection of cognitive impairment.

Despite these limitations, this review makes a significant contribution to the field by systematically mapping the current state of the evidence, identifying key research priorities, and articulating the implications for diverse stakeholders. The findings suggest that NLP could serve as a powerful tool for healthcare systems to improve the early detection and care of cognitive impairment, both at the individual patient level and the population health level. For clinicians, NLP-based screening and decision support integrated into EHR workflows could help prioritize high-risk patients, uncover care gaps, and facilitate evidence-based management. At a system level, combining NLP-derived markers with structured data could enable more precise risk stratification, targeted outreach, and efficient resource allocation.

The reviewed studies highlight several key priorities for advancing the field of NLP for detecting cognitive impairment. First, there is a need for multi-modal platforms that integrate diverse data sources, as demonstrated by Du et al.^24^, who successfully combined NLP features with structured EHR data. Second, applications need to expand beyond screening to include prognostication and trial recruitment, as shown by Wang et al.^23^ in their longitudinal analysis of cognitive decline risk. Third, the field requires better curation of representative expert-annotated corpora, a limitation noted by multiple studies including Gibson et al.^27^ and Hussein et al.^33^. Fourth, researchers should explore advanced feature engineering and temporal modeling techniques to identify the most informative and robust features for early diagnosis and risk stratification. Fifth, standardized reporting guidelines and shared benchmark datasets would facilitate comparisons across studies and accelerate progress toward clinically validated NLP tools. Progress in these areas will be critical for translating the potential of NLP into real-world impact.

Realizing the full potential of NLP for early detection of cognitive decline will require a collaborative and multidisciplinary effort to address the various challenges identified in this review. Future research must also focus on improving model generalizability across diverse populations and healthcare settings, as highlighted by Tyagi et al.^22^, developing more robust approaches to handling documentation variability, as noted by Al-Harrasi et al.^36^, and establishing standardized implementation frameworks, as emphasized by Penfold et al.^21^. Key priorities include establishing annotation standards, defining best practices for model development and validation, implementing explainable and auditable AI systems, and rigorously evaluating the real-world effectiveness and unintended consequences of NLP interventions through pragmatic trials and implementation research. Ethical guidelines must also be established to ensure that NLP innovations are designed and deployed in an equitable and patient-centered manner. Achieving these goals will require close collaboration among clinical, informatics, data science, and patient stakeholders to address the complex sociotechnical barriers to implementation.

As the burden of Alzheimer’s disease and related dementias continues to grow, harnessing the power of NLP and AI to improve early detection and care coordination holds immense promise. This review lays the groundwork for future efforts by synthesizing the current knowledge base, identifying critical gaps and challenges, and charting a roadmap for responsible and impactful research and practice at the intersection of data science, clinical informatics, and brain health.

## Methods

The review followed PRISMA 2020 guidance and was registered in PROSPERO (CRD42024601303)^37^.

### Search strategy

We searched PubMed, Embase, Web of Science, CINAHL, Scopus, MEDLINE, PsycINFO, and the Cochrane Library for studies available from each database’s inception up to September 2024, without language limits. Searches combined terms for: (1) NLP; (2) EHRs/clinical notes; (3) cognitive impairment/dementia; and (4) older adults. The PubMed strategy provided below was adapted across other databases:

((‘natural language processing’ OR ‘computational linguistics’ OR ‘text mining’ OR ‘machine learning’ OR ‘artificial intelligence’ OR ‘speech analysis’ OR ‘language analysis’ OR ‘discourse analysis’ OR ‘linguistic feature’) AND (‘cognitive impairment’ OR ‘cognitive decline’ OR ‘dementia’ OR ‘Alzheimer’ OR ‘mild cognitive impairment’ OR ‘MCI’ OR ‘neurodegenerative’ OR ‘neurological disorder*’) AND (‘clinical notes’ OR ‘electronic health records’ OR ‘EHR’ OR ‘medical documentation’ OR ‘patient records’) AND (‘detection’ OR ‘diagnosis’ OR ‘classification’ OR ‘prediction’ OR ‘screening’ OR ‘early identification’) AND (‘elder’ OR ‘older adult’ OR ‘geriatric’ OR ‘aged’ OR ‘senior’) AND (‘sensitivity’ OR ‘specificity’ OR ‘accuracy’ OR ‘AUC’ OR ‘model performance’ OR ‘evaluation metrics’ OR ‘diagnostic performance’ OR ‘precision’ OR ‘recall’))

We also searched grey literature and consulted experts.

### Eligibility criteria

Included studies met these criteria:

- Population: adults aged ≥60 years at risk of or diagnosed with cognitive impairment, MCI, AD, or other dementias. If age was mixed, ≥80% had to be ≥60 or results were reported separately.
- Intervention: use of NLP methods applied to unstructured EHR clinical notes. Use on other EHR data was allowed if notes were also included.
- Comparator: diagnosis based on cognitive testing, validated criteria, imaging/fluid biomarkers, diagnostic codes, or other reference standards.
- Outcomes: primary—NLP performance metrics (e.g. sensitivity, specificity, F1, AUROC); secondary—NLP methods, target conditions, EHR data, challenges, implementation factors.
- Study design: original research including peer-reviewed articles and preprints.

We excluded studies not using NLP on notes, lacking a reference standard, or without age-stratified results.

### Study selection

A comprehensive literature search across 8 electronic databases yielded 837 initial studies. Records were deduplicated using Covidence, resulting in the removal of 617 duplicate entries (599 identified by Covidence and 18 identified manually). Two independent reviewers screened the remaining 231 titles and abstracts for relevance, with conflicts resolved through discussion or consultation with a third reviewer. Of these, 59 full-text articles were assessed for eligibility. Following detailed review, 41 studies were excluded based on predefined criteria. The final analysis included 18 studies that met all inclusion criteria and specifically investigated cognitive decline using natural language processing of electronic health record clinical notes in older adults.

### Data extraction

An extraction form was piloted on five studies. Two authors independently extracted data from each study, with disagreements arbitrated by consensus. Study authors were contacted about unclear information. The extraction covered ten comprehensive domains:

Study characteristics included first author, publication year, country, study design (e.g., retrospective cohort, case-control), study setting (e.g., primary care, specialty clinic), sample size, and study duration. Population characteristics encompassed age distributions, sex ratios, race/ethnicity demographics, education levels, cognitive status categorizations (normal, MCI, dementia subtypes), comorbidities, and medication use patterns.

For EHR data, we documented system details (vendor, product), clinical note types utilized (progress notes, consults, discharge summaries), time period of data extraction, and preprocessing steps. Reference standard information included cognitive assessment tools, diagnostic criteria (e.g., DSM-5, Petersen criteria for MCI), and the process of standard application.

NLP methodological details covered preprocessing techniques, specific NLP tasks and tools employed (named entity recognition, parsing, relation extraction), knowledge sources, clinical features generated, and software specifications. Machine learning methods were characterized by algorithms used, feature selection approaches, training/validation strategies, and hyperparameter optimization procedures.

Performance metrics extraction focused on reported outcomes (AUROC, sensitivity, specificity, F1 score), metric definitions, and statistical significance. Error analysis documentation included false positive/negative patterns and limitation assessments. Implementation considerations encompassed computational efficiency, model interpretability, generalizability evidence, and workflow integration approaches.

Additional extracted information included funding sources, conflict of interest declarations, ethics approvals, and data-sharing statements. This comprehensive extraction framework enabled systematic comparison across studies while capturing both technical and practical aspects of NLP implementation for cognitive decline detection.

## Supplementary information

Checklist

## Data Availability

The datasets analyzed in this systematic review are available from the cited publications.

## Abbreviations

AD: Alzheimer’s disease
ADEPt: Alzheimer’s Disease Extraction Pipeline
AI: artificial intelligence
AUC: area under the curve
AUROC: area under the receiver operating characteristic curve
CAD: coronary artery disease
CI: cognitive impairment/confidence interval (context dependent)
CINAHL: Cumulative Index to Nursing and Allied Health Literature
CRIS: Clinical Record Interactive Search
CT: computed tomography
CV: cross-validation
DW: data warehouse
EHR: electronic health record
EMRPC: electronic medical record primary care
F1: F1 score (harmonic mean of precision and recall)
FINGER: Finnish Geriatric Intervention Study to Prevent Cognitive Impairment and Disability
HTN: hypertension
*I*^2^: *I* squared (measure of statistical heterogeneity)
IQR: interquartile range
MCI: mild cognitive impairment
MEDLINE: medical literature analysis and retrieval system online
MIMIC-III: Medical Information Mart for Intensive Care III
ML: machine learning
MMSE: Mini-Mental State Examination
MRI: magnetic resonance imaging
N/A: not applicable/not available
NLP: natural language processing
PRISMA: Preferred Reporting Items for Systematic Reviews and Meta-Analyses
PRISMA-S: PRISMA search extension
PROSPERO: International Prospective Register of Systematic Reviews
QUADAS-2: Quality Assessment of Diagnostic Accuracy Studies-2
RF: random forest
Rep.: reported
Sens: sensitivity
SNOMED CT: Systematized Nomenclature of Medicine – Clinical Terms
Spec: specificity
TBI: traumatic brain injury
UK: United Kingdom
UMLS: Unified Medical Language System
US/USA: United States/United States of America
VA: Veterans Affairs
